# Prebiotic potential and metabolic benefits of *Acorus gramineus* rhizome-derived polysaccharides in a high-fat diet-induced obesity model

**DOI:** 10.3389/fnut.2025.1605201

**Published:** 2025-06-10

**Authors:** Miri Park, Yu-Rim Chae, Hye-Bin Lee, Jang-Eun Lee, Yu Ra Lee, Jinyoung Hur, Ho-Young Park

**Affiliations:** ^1^Food Functionality Research Division, Korea Food Research Institute, Wanju-gun, Republic of Korea; ^2^Food Convergence Research Division, Korea Food Research Institute, Wanju-gun, Republic of Korea; ^3^Department of Food Biotechnology, University of Science and Technology, Daejeon, Republic of Korea

**Keywords:** *Acorus gramineus*, polysaccharides, metabolic dysfunction-associated steatotic liver disease, metabolic endotoxemia, prebiotic

## Abstract

Metabolic dysfunction-associated steatotic liver disease (MASLD) is a global health issue that causes severe liver abnormalities and metabolic dysregulation, incurring substantial costs to healthcare systems. *Acorus gramineus*, a traditional remedy, has been studied for its anti-inflammatory and neuroprotective effects. However, its potential role in the management of metabolic disorders via its polysaccharide components remains unclear. This study aimed to investigate the effects of the *A. gramineus* rhizome-derived polysaccharide (AGRP) on prebiotic potential and metabolic health in a high-fat diet-induced obesity mouse model. AGRP showed prebiotic efficacy by targeting representative intestinal microorganisms and demonstrated the ability to promote butyrate production. Importantly, mice fed a high-fat diet supplemented with AGRP showed significant improvements in metabolic disease markers, including reductions in body fat and white adipose tissue mass, compared to those in controls. Additionally, serum metabolic analysis of AGRP-administered mice demonstrated positive changes in liver enzymes, lipid profile, and glucose metabolism, as well as reduced lipid accumulation and downregulation of lipogenic genes. AGRP increased the intestinal barrier function by modulating endotoxins and tight junction protein expression, demonstrating its interaction with improved intestinal health. These findings suggest that AGRP is a promising prebiotic with potential applications in the management of MASLD and related metabolic disorders.

## 1 Introduction

Metabolic dysfunction-associated steatotic liver disease (MASLD) has emerged as one of the most prevalent liver disorders worldwide, affecting a substantial portion of the adult population due to its close association with obesity and metabolic syndrome (1). MASLD encompasses a range of liver abnormalities, from simple hepatic steatosis, where excess lipids accumulate in liver cells, to non-alcoholic steatohepatitis (NASH), a more severe form characterized by liver inflammation and fibrosis. If left unmanaged, NASH can progress to cirrhosis or hepatocellular carcinoma, leading to significant health and economic burdens globally (2). Given the limited therapeutic options currently available for MASLD, researchers have focused on preventive and non-invasive strategies to curb disease progression, particularly through dietary interventions that target the underlying metabolic dysfunctions (3).

The gut-liver axis, representing the bidirectional relationship between the gut microbiota and liver function, has been increasingly recognized as a key player in MASLD pathogenesis (4). This relationship hinges on the role of gut microbiota in regulating immune responses, nutrient metabolism, and intestinal barrier integrity. Dysbiosis, characterized by an overabundance of gram-negative pathogenic bacteria, increases the production of lipopolysaccharides (LPS), a major component of their outer membrane. This microbial imbalance impairs the expression of tight junction proteins and compromises the mucosal barrier, leading to increased intestinal permeability. As a result, LPS can translocate from the gut lumen into the systemic circulation, a condition known as metabolic endotoxemia, which promotes systemic inflammation and contributes to metabolic disorders such as MASLD (5–7).

Prebiotics have emerged as a promising intervention for restoring gut microbiota balance and, consequently, mitigating the effects of metabolic endotoxemia in MASLD. Prebiotics are indigestible dietary fibers that selectively stimulate the growth and activity of beneficial gut bacteria, particularly Bifidobacterium and Lactobacillus species, which play a protective role in maintaining intestinal integrity and suppressing pathogenic microbes (8, 9). Recent studies indicate that prebiotics can modulate gut microbial composition and function, improving systemic metabolic markers such as blood lipid profiles, glucose homeostasis, and inflammatory cytokine levels. Such effects position prebiotics as viable candidates for addressing the metabolic and inflammatory components of MASLD (6).

*Acorus gramineus* rhizome (AGR), a medicinal plant traditionally used in East Asia for its neuroprotective and cognitive-enhancing effects, present a novel approach to MASLD management. Recent studies have shown that AGR contains bioactive compounds, including polysaccharides, which may have beneficial effects on gut health and metabolism (10). Previous research on *A. gramineus* has predominantly focused on its effects on neural health, including its roles in alleviating cognitive decline and providing antioxidant protection against neurodegeneration (11, 12). However, emerging evidence suggests that the AGR has been reported to possess anti-inflammatory and antioxidant properties that may be relevant to its potential role in managing metabolic disorders (13).

The objective of this study is to evaluate the effects of AGRP on gut microbiota composition, intestinal barrier function, and hepatic lipid metabolism using a high-fat diet-induced model of metabolic disease. By elucidating these mechanisms, we aim to provide new insights into the potential of AGRP as a dietary intervention for MASLD. This research seeks to bridge the gap between traditional uses of *A. gramineus* and its emerging role in metabolic health, proposing a novel preventive strategy that targets both gut and liver health in the context of metabolic disease.

## 2 Materials and methods

### 2.1 Preparation of *Acorus gramineus* rhizome-derived polysaccharide

The rhizomes of *A. gramineus* were harvested, thoroughly washed, and air-dried before extraction. Approximately 200 g of dried rhizomes were finely ground and extracted by refluxing with 20 times the volume of distilled water at 80°C for 3 h. The extract was filtered through Whatman No. 4 filter paper, then concentrated to 1/10 of its original volume using a rotary vacuum evaporator (R-114; Buchi Labortechnik, Flawil, Switzerland). To precipitate the polysaccharides, four times the volume of 95% ethanol was added, and the solution was left to incubate at 4°C for 16 h. The precipitate was collected by centrifugation at 3,000 × g for 30 min, followed by dialysis at 4°C for 48 h (molecular weight cutoff: 12–14 kDa; Spectrum Laboratories, Rancho Dominguez, CA, United States). The resulting product, AGRP, was obtained by freeze-drying the concentrate.

### 2.2 Composition analysis and of AGRP

AGRP’s total carbohydrate content was evaluated using the phenol-sulfuric acid method, utilizing glucose as a standard (14). Monosaccharide composition was analyzed by gas chromatography following hydrolysis with 2 M trifluoroacetic acid at 120°C for 90 min. The hydrolyzed sample was separated into neutral sugars and uronic acids using a Sep-pak QMA cartridge (Waters, Dublin, Ireland). Gas chromatography (ACME-6100, Young-Lin, Anyang, Republic of Korea) with a flame ionization detector and SP-2380 capillary column (Supelco, Bellefonte, PA, United States) was used to quantify monosaccharide components.

### 2.3 Prebiotic activity assay

Prebiotic activity assays were performed as described previously (15). Prebiotic activity was assessed to evaluate AGRP’s ability to promote the growth of beneficial gut bacteria. Three commercial probiotic strains—*Lactiplantibacillus plantarum* (*L. plantarum* MG989), *Lactobacillus delbrueckii* subsp. *bulgaricus* (*L. bulgaricus* MG5165), and *Bifidobacterium longum* (*B. longum* MG723)—were obtained from Mediogen (Jecheon, Republic of Korea). Additionally, *Escherichia coli* (*E. coli* KCTC2441) was included as a representative enteric bacterium. Bacterial strains were cultured on appropriate media: De Man-Rogosa-Sharpe (MRS) agar and broth for *Lactobacillus* and *Bifidobacterium* strains, and tryptic soy agar and broth for *E. coli*. Cultures were grown at 37°C for 24 h, centrifuged at 1,000 × g for 5 min, and resuspended in M9 broth supplemented with 0.1% glucose, 0.0015% CaCl₂, and 0.05% MgSO_₄_.

The prebiotic effect of AGRP was evaluated by inoculating bacterial cultures with glucose (5,000 μg/mL), inulin (5,000 μg/mL, positive control), and AGRP at two concentrations (0.1% AGRP and 0.5% AGRP in M9 broth) for 24 h at 37°C. M9 minimal broth was supplemented with 2 g/L glucose, 0.015 g/L CaCl₂, and 0.5 g/L MgSO_₄_. This basal medium contains no complex carbon or nitrogen sources, ensuring that any observed growth reflects the effect of the added polysaccharide or control compounds. Bacterial growth was monitored by measuring optical density at 600 nm. Prebiotic activity scores were calculated to quantify the selective growth-promoting effect of AGRP on probiotics relative to enteric bacteria.


$$\begin{array}{l} \text{Prebiotic activity score}=\\ \frac{\begin{array}{l} \text{probioticlog}\;\text{CFU}/\text{mLon the prebiotic}\;\text{at}\;24\;h-\\ \text{probioticlog}\;\text{CFU}/\text{mLon the prebiotic}\;\text{at}\;0\;h \end{array}}{\begin{array}{l} \text{probioticlog}\;\text{CFU}/\text{mLon the glucose}\;\text{at}\;24\;h-\\ \text{probioticlog}\;\text{CFU}/\text{mLon the glucose}\;\text{at}\;0\;h \end{array}}-\\ \frac{\begin{array}{l} \text{entericlog}\;\text{CFU}/\text{mLon the prebiotic}\;\text{at}\;24\;h-\\ \text{entericlog}\;\text{CFU}/\text{mLon the prebiotic}\;\text{at}\;0\;h \end{array}}{\begin{array}{l} \text{entericlog}\;\text{CFU}/\text{mLon the glucose}\;\text{at}\;24\;h-\\ \text{entericlog}\;\text{CFU}/\text{mLon the glucose}\;\text{at}\;0\;h \end{array}} \end{array}$$

### 2.4 Analysis of medium butyrate content

Probiotic strains were cultured in M9 broth supplemented with 0.1% or 0.5% AGRP and incubated anaerobically at 37°C for 24 h. Following fermentation, the culture supernatant was collected by centrifugation and used for butyrate analysis. Butyrate contents of bacterial broth were analyzed through a headspace solid-phase microextraction GC-MSMS system (7890A GC with 7010C triple quadrupole tandem mass spectrometry, Agilent, Santa Clara, CA, United States) using CAR/PDMS 85 μm SPME fiber (Supelco, Bellefont, PA, United States) and DB WAXetr capillary column (0.25 mm × 30 m, 0.25 μm film thickness, Agilent). The analysis was in splitless injection mode, and helium was used as a carrier gas at a constant flow of 1 mL/min. The optimal analytical condition was starting at 80°C for 2 min, increased to 100°C/min, ramped to 130°C at the rate of 5°C/min, followed by 160°C at the rate 10°C/min and 220°C at the rate of 20°C/min, and finally held 5 min. The temperature of MS transfer line was 240°C, and the EI source and quad were kept at 230°C and 150°C. The triple quadrupole was operated with N_2_ as the collision gas at a flow of 1.5 mL/min and He as the quenching gas at a 2.25 mL flow. The multiple reaction monitoring (MRM) specifications of butyric acid and internal standard were represented in Supplementary Table S1.

### 2.5 Animal experiment

#### 2.5.1 Mice and diet

The Institutional Animal Care and Use Committee of the Korea Food Research Institute approved the experimental animal protocol (IACUC approval number: KFRI-M-22038). Seven-week-old male C57BL/6 mice were procured from G-Bio (Gwangju, Republic of Korea) and acclimated to a controlled environment (23 ± 2°C, 60 ± 5% humidity, 12-h light/dark cycle) for 1 week with ad libitum access to sterilized water and a standard AIN-93G diet (Dyets, Bethlehem, PA, United States). After acclimation, mice were randomly assigned to the following groups: a normal diet (ND; AIN-93G), high-fat diet (HFD; 60% kcal fat), HFD with 5 mg/kg fructooligosaccharide (FOS, positive control), HFD with 5 mg/kg AGRP (AGRP5), and HFD with 10 mg/kg AGRP (AGRP10). AGRP and FOS were administered orally once daily for 8 weeks. Food intake and body weight were recorded weekly, and at the end of the experimental period, body composition and adipose tissue weight were assessed using imaging scans (InAlyzer, Medikors Inc., Seongnam, South Korea).

#### 2.5.2 Oral glucose tolerance tests analysis

After a 14-h fast, the mice were administered glucose (Sigma-Aldrich, St. Louis, MO, United States) (2 g/kg body weight) orally, and the glucose levels in tail vein blood were measured using a test strip and glucometer (Accu-Chek Performa; Roche Diagnostics, Indianapolis, IN, United States) at 0, 30, 60, 90, and 120 min following glucose administration.

#### 2.5.3 Myeloperoxidase activity analysis

Myeloperoxidase (MPO) activity was assessed using a previously published method (15), with some minor modifications. Approximately 50 mg of fresh colon tissue was homogenized in an ice-cold 50 mM sodium phosphate buffer with 0.5% hexadecyltrimethyl ammonium bromide. The homogenate was frozen at −70°C for 3 h, then centrifugated at 12,000 × *g* for 15 min at 4°C. Following three centrifugations, 400 μL of a solution comprising 50 mM sodium phosphate buffer, 0.129 mg/mL O-dianisidine, and 0.0005% H2O2 was mixed with 100 μL of the supernatant. The absorbance of the final mixture was measured at 492 nm.

#### 2.5.4 Blood biochemical analysis

Blood samples were collected after an overnight fast, and serum was separated by centrifugation. Serum samples were analyzed for liver enzyme activities (ALT, AST), lipid profile (total cholesterol, HDL-C, LDL-C, and triglycerides), and insulin levels using commercial assay kits (Thermo Fisher Scientific, Waltham, MA, United States). Insulin resistance was measured using the homeostatic model assessment of insulin resistance (HOMA-IR).


$$\text{HOMA}-\text{IR}=(\begin{array}{l} \text{fasting insulin}\;(\mathrm{\mu U}/\text{mL})\times \\ \text{fasting glucose}\;(\text{mg}/\text{dL}) \end{array})/405$$

#### 2.5.5 Histological analysis

Histological analyses of liver tissues were conducted using Oil Red O staining to assess lipid accumulation and hematoxylin and eosin (H&E) staining to evaluate general tissue morphology. Liver, epididymal white adipose tissue (eWAT), and colon samples were fixed in 10% formalin, embedded in paraffin, and sectioned for staining. Image analysis was performed to quantify lipid content and assess cellular architecture changes.

#### 2.5.6 Analysis of protein expression in colon tissue

Liver, colon, and eWAT tissues were lysed in a protein extraction solution (PRO-PREP, Intron Biotechnology, Korea), and the protein concentration was quantified using a Bio-Rad protein assay (Bio-Rad, Hercules, CA, United States). The proteins were separated by electrophoresis on a 15% sodium dodecyl sulfate polyacrylamide gel. After being moved onto polyvinylidene difluoride membranes, the proteins were blocked for 1 h using 5% skim milk. They were incubated together overnight at 4°C using the following primary antibodies (Abcam, Cambridge): acetyl-CoA carboxylase (ACC; ab72046), fatty acid synthase (FAS; ab22759), tumor necrosis factor-alpha (TNF-*α*; ab6671), cluster of differentiation 36 (CD36; ab133625), monocyte chemoattractant protein-1 (MCP1; ab25124), interleukin-6 (IL-6; ab6672), sterol regulatory element-binding protein 1 (SREBP1; ab191857), *β*-actin (ab8224), carbohydrate-responsive element-binding protein (ChREBP; 58069), and stearoyl-Coenzyme A desaturase 1 (SCD1; 2794). After reacting Abcam secondary antibodies, including HRP-conjugated anti-rabbit antibody and anti-mouse antibody, the intensity of the developed band was confirmed using a EZ Western Lumi Femto Kit (DoGenBio Co. Ltd., Seoul, Republic of Korea) for chemiluminescence detection using the ChemiDoc XRS + imaging system (Bio-Rad). For each sample, *β*-actin was used as an internal control.

### 2.6 Statistical analysis

All data are expressed as mean ± standard deviation (SD). Statistical analyses were conducted based on a randomized group design with five experimental groups (*n* = 9 per group), as described in the animal study section. One-way analysis of variance (ANOVA) was used to evaluate group differences, followed by Tukey’s post-hoc test for multiple comparisons. For prebiotic activity scores, Duncan’s multiple range test was applied. A *p*-value of <0.05 was considered statistically significant. Data analysis was performed using IBM SPSS Statistics (version 20; IBM Corp., Armonk, NY, United States).

## 3 Results

### 3.1 Chemical composition of AGRP

The chemical composition was analyzed to identify structural components relevant to AGRP’s biological activity. The sugar composition of AGRP revealed a complex and diverse profile of monosaccharides, each with potential physiological significance (Table 1). The predominant sugar is galacturonic acid (64.8 mg/g), followed by arabinose (56.9 mg/g), galactose (42.9 mg/g), xylose (37.7 mg/g), and glucose (30.0 mg/g). These constituent sugars are commonly found in pectins and arabinogalactans, which are known to modulate the immune system and promote the growth of beneficial gut bacteria. Their presence suggests that AGRP possesses the fermentable characteristics required for prebiotic function (16–18).

**Table 1:** Chemical compositions of *Acorus gramineus* rhizome-derived polysaccharides (AGRP).

	Value (mg/g)
Total carbohydrate	69.2 ± 4.1
Monosaccharides	
Mannose	8.1 ± 0.1
Rhamnose	25.2 ± 0.3
Glucuronic acid	14.1 ± 1.8
Galacturonic acid	194.4 ± 9.4
Glucose	90.0 ± 2.1
Galactose	128.7 ± 3.4
Xylose	113.1 ± 0.9
Arabinose	170.7 ± 2.4
Fucose	N.D.

N.D., not detected.

### 3.2 AGRP shows enhancing growth of beneficial gut bacteria

**Figure 1:**
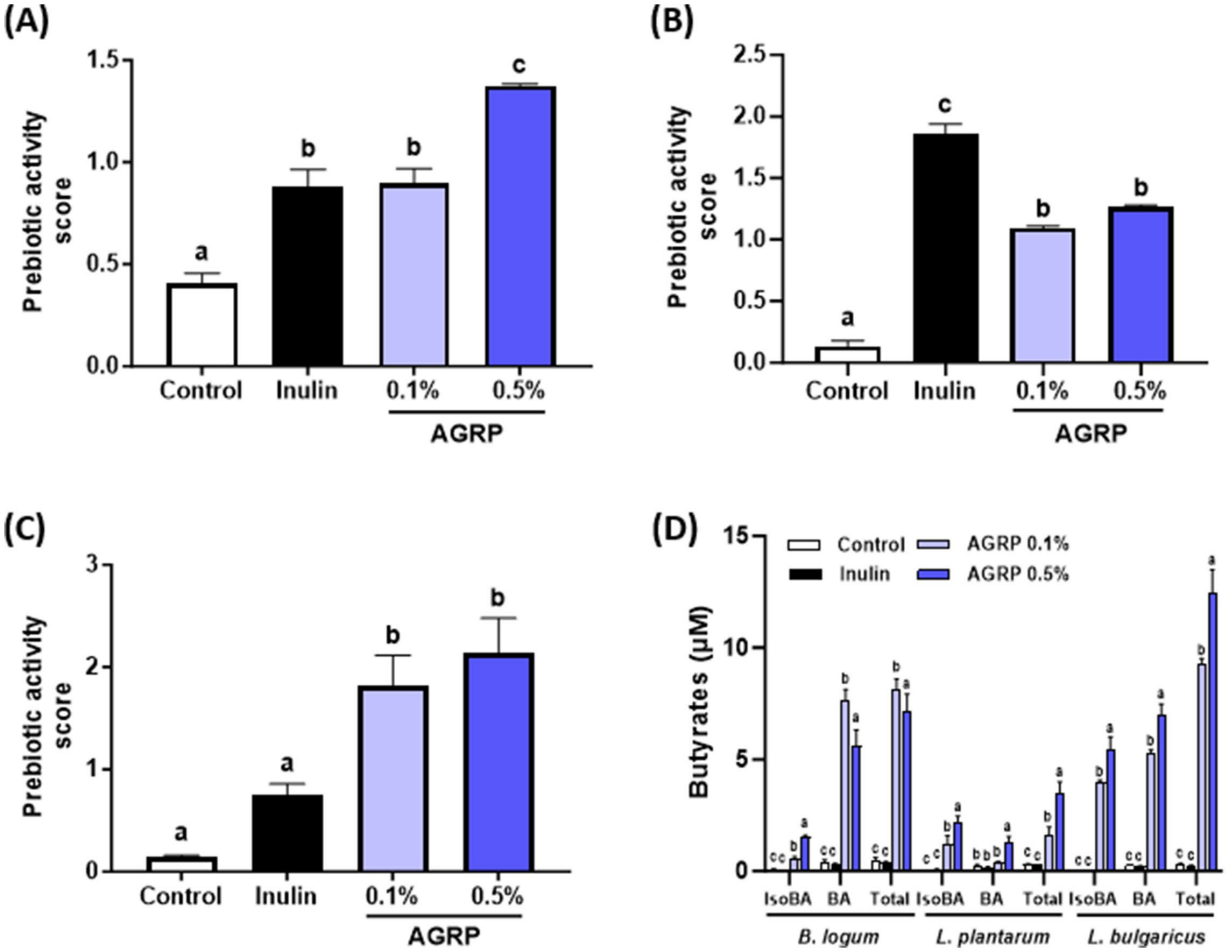
*Acorus gramineus* rhizome-derived polysaccharides (AGRP) enhances beneficial gut bacteria growth and butyrate production. **(A)** *Bifidobacterium longum*; **(B)** *Lactobacillus plantarum*; **(C)** *Lactobacillus delbrueckii* subsp. *bulgaricus*; **(D)** Butyrate production using three probiotic strains. Panels **(A–C)** show bacterial growth (OD600), not butyrate levels. Prebiotic activity scores were calculated as described. Inulin was used as a positive control. All data are expressed as mean ± standard error of the mean (*n* = 3). According to Duncan **(A–C)** and Tukey **(D)** multiple range tests, values with different letters are significantly different at *p* < 0.05.

In this study, we evaluated the prebiotic activity of AGRP using four beneficial bacterial species, focusing on *Lactobacillus* and *Bifidobacterium* strains, which are known to have positive effects on the gut microbiome. Bacterial growth was negligible in M9 broth alone, confirming it provided no sufficient nutrient support. In contrast, significant proliferation occurred upon addition of AGRP or inulin, indicating that the prebiotic effect was due solely to the polysaccharide sample. The results show that AGRP exhibited significant prebiotic activity, especially at 0.5% concentration (Figures 1A–C). The calculated prebiotic activity scores revealed that *L. bulgaricus* had the highest score (2.8), followed by *B. longum* (1.5), whereas *L. plantarum* showed minimal response compared to the inulin control. These results highlight the selective ability of AGRP to promote the growth of specific probiotic strains. In particular, *L. bulgaricus* showed marked growth with up to 2.8 times higher bacterial growth rate than that of the positive control, inulin, but no substantial change was observed in the abundance of *L. plantarum*. The study also found that both types of *Bifidobacterium* exhibited growth levels 1.5 times higher than those observed with inulin at a concentration of 0.5% AGRP. The differential growth responses observed in the tested bacterial strains suggested that AGRP may have a selective prebiotic effect. This selectivity is a desirable property of prebiotics, as it allows the targeted regulation of gut microbiota composition.

### 3.3 AGRP increases butyrate production by probiotic strains

The concentrations of isobutyric, butyric, and total butyric acid generated during the 24 h fermentation of AGRP by the probiotic strains are shown in Figure 1D. The AGRP groups (0.1 and 0.5%) exhibited significantly higher levels of isobutyric, butyric, and total butyric acids than in the control and inulin groups. Moreover, higher concentrations of AGRP produced more short chain fatty acids in *L. plantarum* and *L. bulgaricus*. These findings indicated that cultures utilizing AGRP as the carbon source yielded altered butyrate profiles compared to that of the control group for all probiotic strains. In particular, AGRP’s ability of AGRP to stimulate the growth of beneficial bacteria at levels exceeding those of inulin (a well-known prebiotic) indicates its potential as a novel natural source-derived prebiotic ingredient.

### 3.4 AGRP improve high-fat-diet-induced metabolic disorder parameters

**Figure 2:**
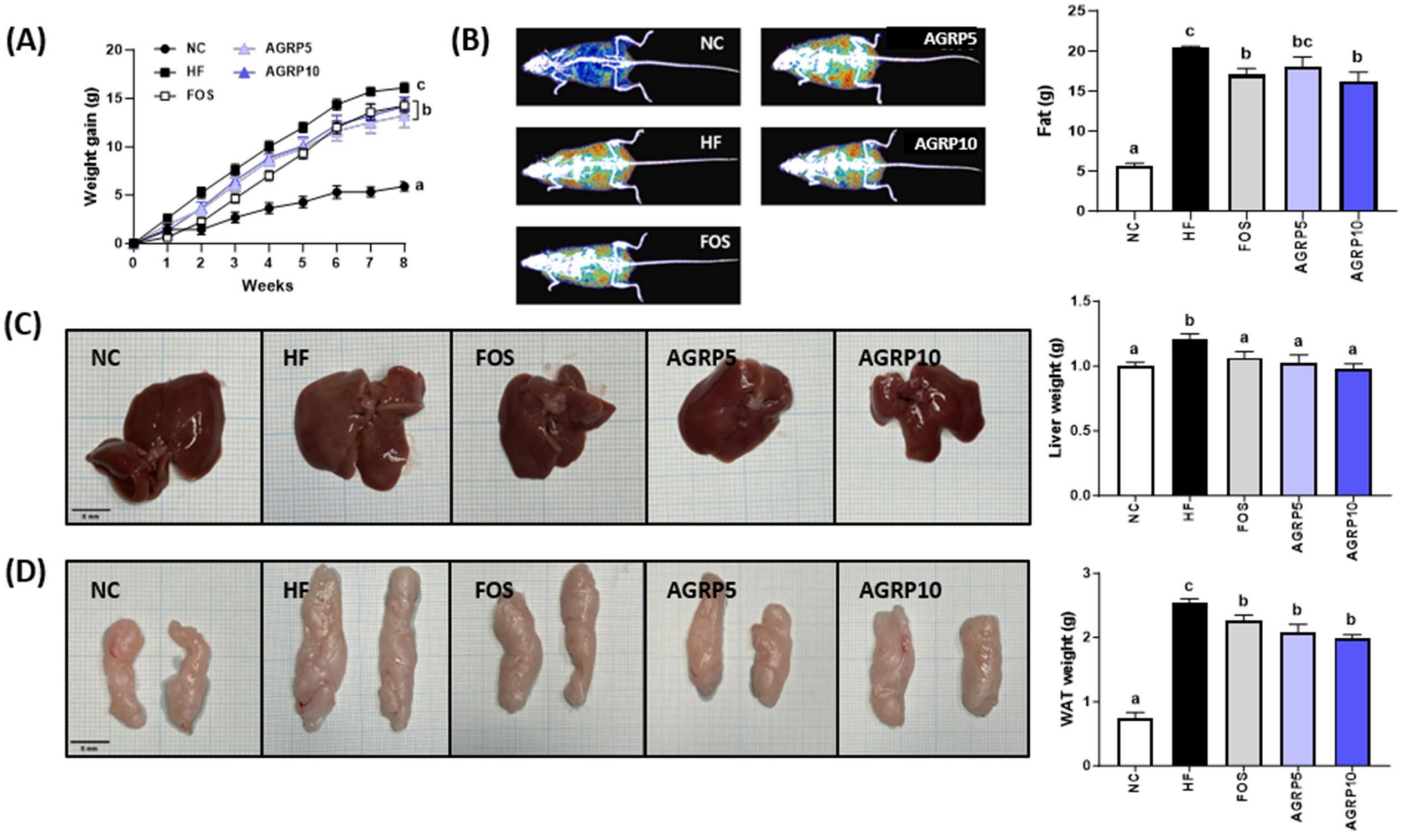
*Acorus gramineus* rhizome-derived polysaccharides (AGRP) reduces fat accumulation. **(A)** Changes in weight increase over the trial period; **(B)** Images of body composition and fat grams; **(C)** Images of the liver and weight; and **(D)** Images of the liver WAT and weight. **(C,D)** Scale bars are 5 mm. All data are presented as mean ± standard error of the mean (*n* = 9). Values with different letters are significantly different at *p* < 0.05 by Duncan’s multiple range tests. ND, normal diet; HF, high-fat diet; FOS, fructooligosaccharide.

The group fed AGRP for 8 weeks showed significantly reduced body weight gain compared to that of the HFD group (Figure 2A). Although both AGRP doses were effective, no significant difference was observed between low and high concentrations.

Body composition analysis using an InAlyzer showed that AGRP feeding reduced fat content in HFD mice (Figure 2B). In addition, AGRP administration decreased both liver and epididymal white adipose tissue (eWAT) sizes compared to those in the HFD group (Figures 2C,D). Taken together, these findings indicate that AGRP significantly reduced body weight gain, liver weight, and fat accumulation in diet-induced obese mice.

### 3.5 AGRP improves serum lipid profile and reduces insulin resistance

**Figure 3:**
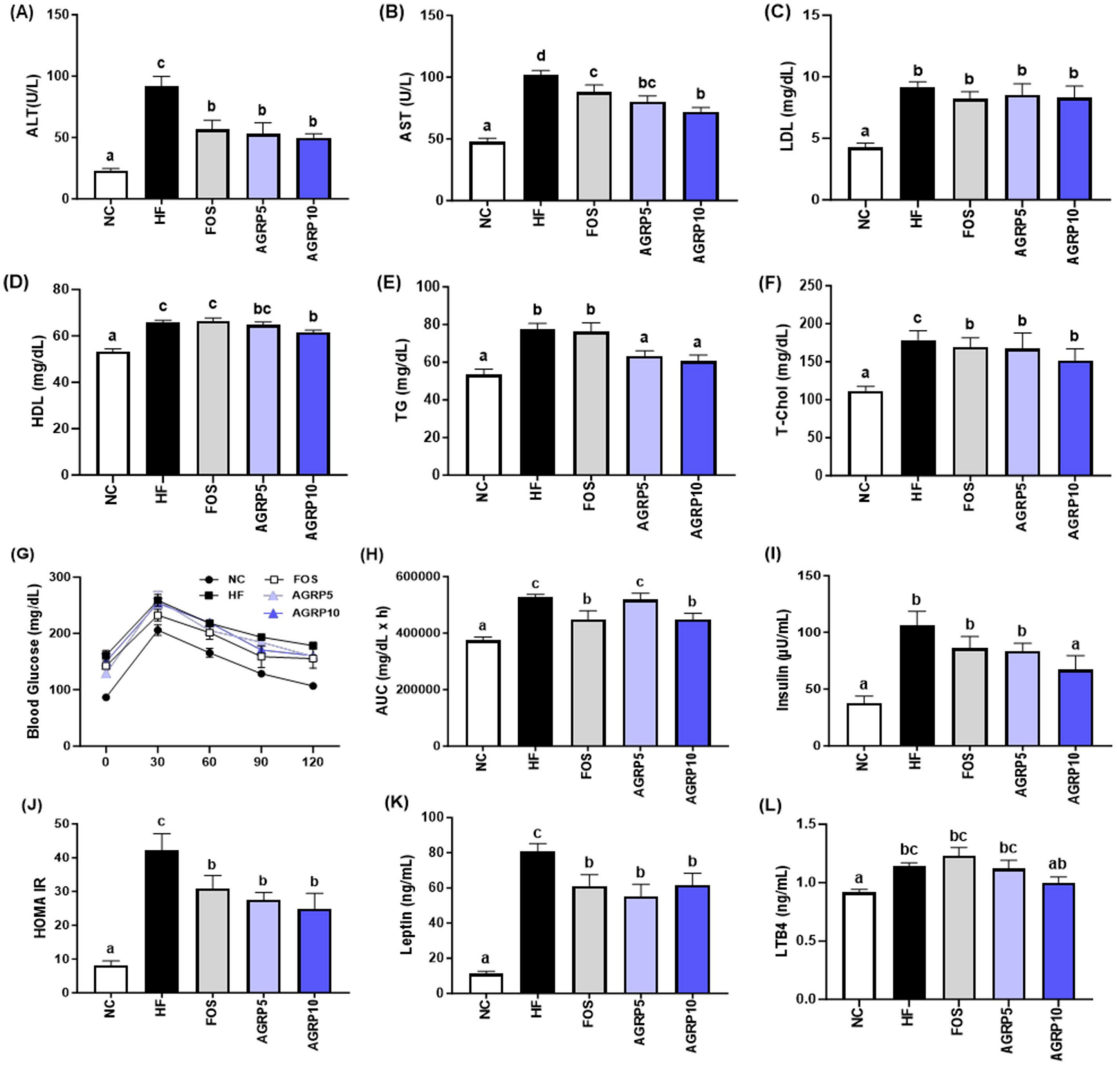
*Acorus gramineus* rhizome-derived polysaccharides (AGRP) improves high-fat-diet-induced serum lipid profiles and reduces insulin resistance. **(A)** Serum alanine aminotransferase (ALT); **(B)** Serum aspartate aminotransferase (AST); **(C)** LDL cholesterol **(D)** Serum HDL cholesterol; **(E)** Serum triglyceride (TG); **(F)** Serum total cholesterol. **(G,H)** Blood glucose levels during oral glucose tolerance tests and area under the curve of blood glucose levels; **(I)** Serum insulin levels; **(J)** Homeostatic model assessment for insulin resistance (HOMA-IR) index; **(K)** Leptin; **(L)** LTB4. All data are presented as mean ± standard error of the mean (*n* = 9). Values with different letters are significantly different at *p* < 0.05 by Duncan’s multiple range tests. NC, normal control diet; HF, high-fat diet; FOS, fructooligosaccharide.

To evaluate the effect of AGRP on serum lipid profile, the serum levels of ALT, AST, TG, TC, HDL, and LDL were measured. The serum ALT, AST, HDL, TG, and TC levels of mice in the AGRP treatment group were significantly lower than those in the HFD group; however, the LDL cholesterol levels were not significantly different (*p* < 0.05, Figures 3A–F). Other parameters did not show significant differences between AGRP doses.

The OGTT and insulin resistance are recognized as important pathophysiological factors in MASLD. We performed glucose tolerance and insulin resistance experiments to investigate the relationship between AGRP administration and fatty liver disease in HFD-induced mice. AGRP10 administration significantly reduced glucose intolerance in HFD mice (*p* < 0.05, Figures 3G–I).

Additionally, AGRP significantly decreased homeostatic assessment of insulin resistance (HOMA-IR) scores, as well as leptin and LTB4, markers involved in immunity and inflammation in HFD mice (Figures 3J–L).

### 3.6 AGRP reduces steatosis and inflammation in MASLD

**Figure 4:**
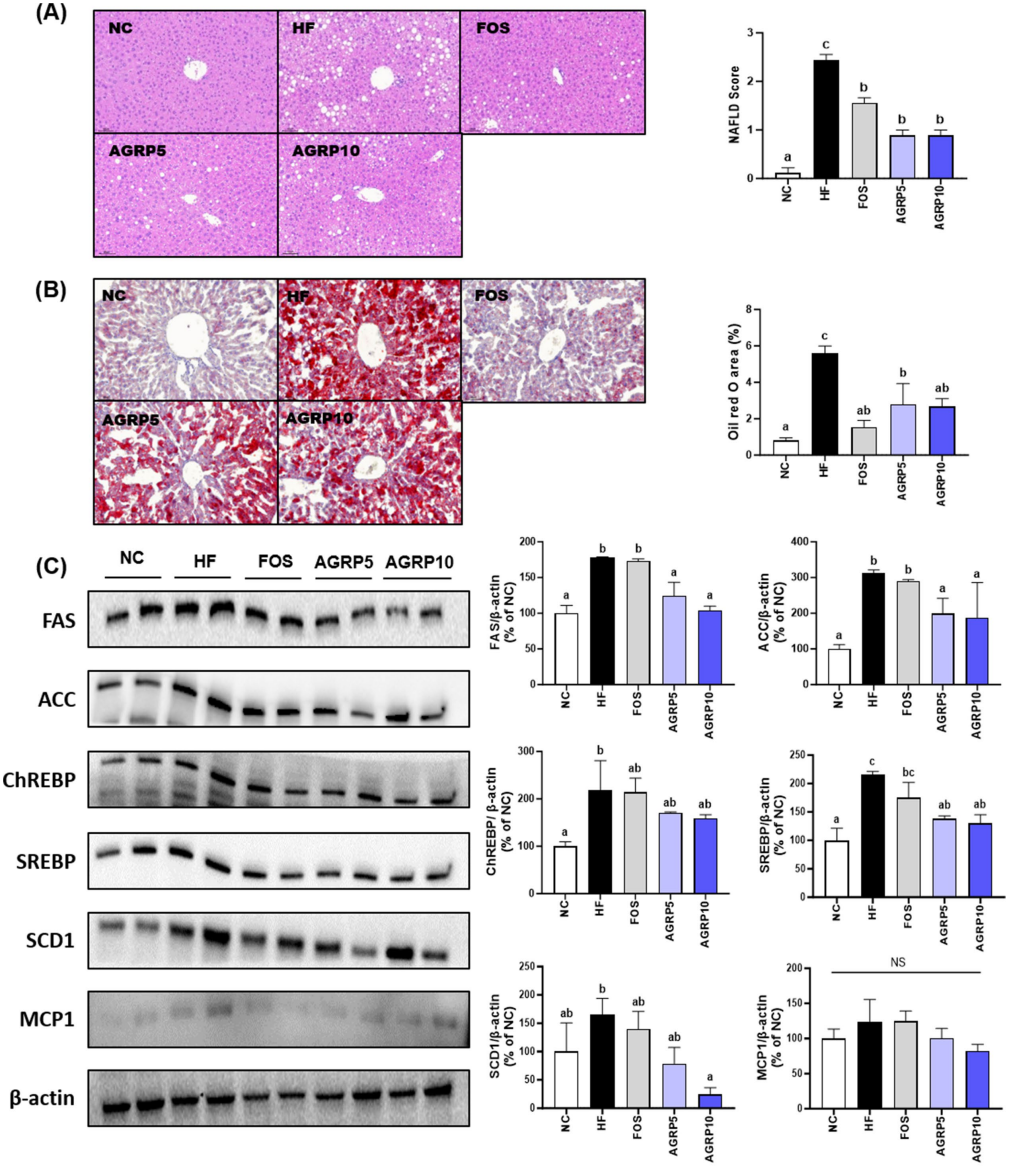
*Acorus gramineus* rhizome-derived polysaccharides (AGRP) reduces steatosis and inflammation in metabolic dysfunction-associated fatty liver disease (MASLD). **(A)** Hematoxylin end eosin (H&E) staining and quantification of the MASLD score in liver tissues; **(B)** Oil Red O (ORO) staining; **(C)** representative western blots of lipid metabolism-related proteins, including fatty acid synthase (FAS), acetyl-CoA carboxylase (ACC), sterol regulatory element-binding protein 1 (SREBP1), carbohydrate-responsive element-binding protein (ChREBP), stearoyl-CoA desaturase 1 (SCD1), and monocyte chemoattractant protein 1 (MCP1) in the liver. The mean ± standard error of the mean (*n* = 3) is used to represent all data. According to Duncan’s multiple range tests, values with different letters are substantially different at *p* < 0.05. NC, normal control diet; HF, high-fat diet; FOS, fructooligosaccharide.

H&E staining of the liver tissue was performed to determine the effect of AGRP on steatosis and lobular inflammation in hepatocytes. The levels of steatosis and inflammation were lower in the AGRP group than in the HFD group, indicating that NAS was significantly reduced by AGRP administration (*p* < 0.05; Figure 4A). Additionally, AGRP administration significantly inhibited HFD-induced lipid accumulation (*p* < 0.05; Figure 4B). The expression levels of the proteins involved in lipid metabolism were evaluated to determine the inhibitory efficacy of AGRP on hepatic fat accumulation. AGRP intake downregulated the expression of lipid metabolism-related proteins compared to that in the HFD group, but there was no significant difference in CHREBP and MCP-1 levels (Figure 4C). In summary, the AGRP administration group showed a decrease in the expression of lipogenesis-related proteins compared to that of the HFD group, indicating that AGRP alleviates HF-induced fatty liver symptoms.

### 3.7 AGRP reduces adipocyte size and lipogenesis in eWAT

**Figure 5:**
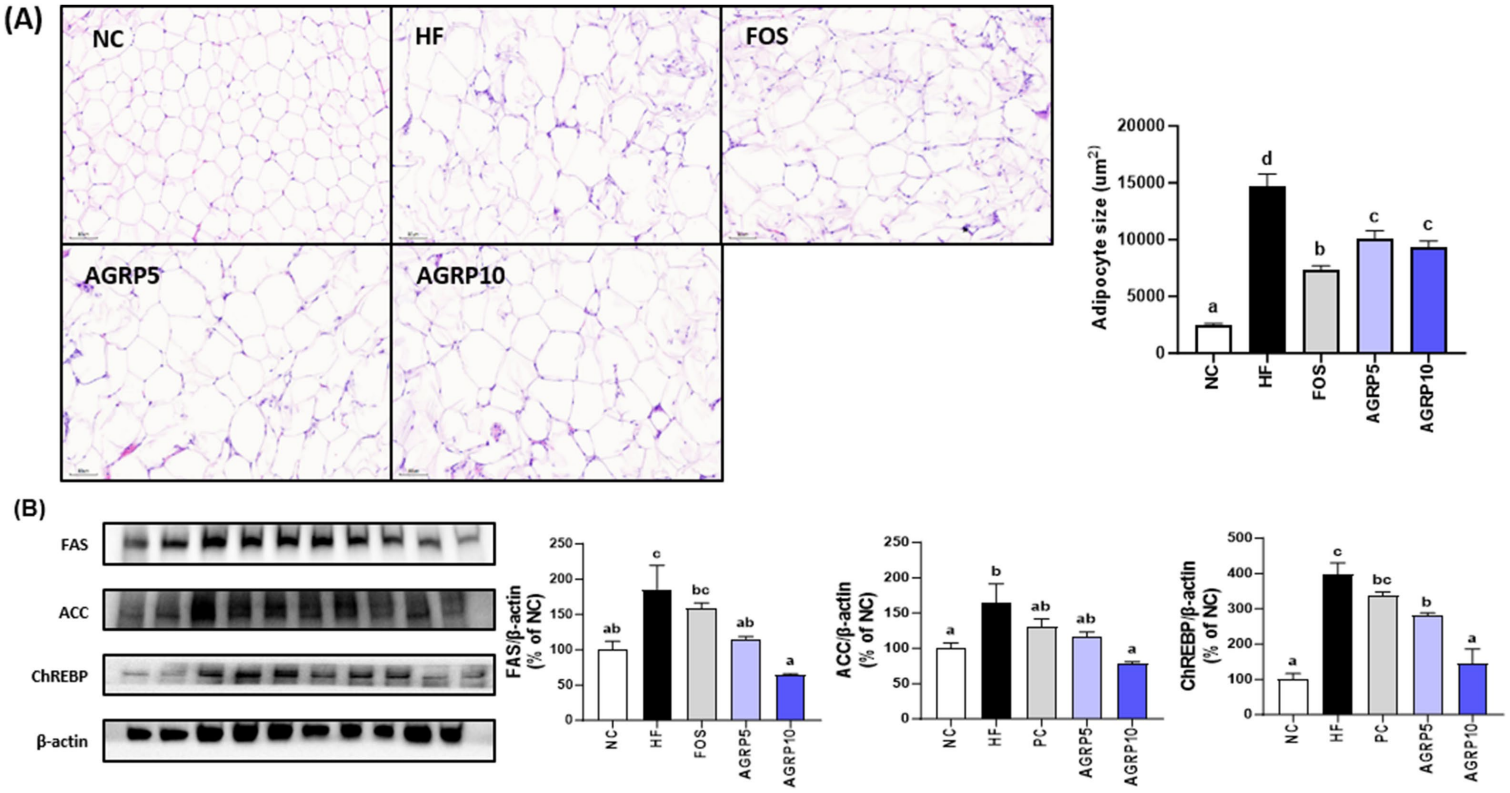
*Acorus gramineus* rhizome-derived polysaccharides (AGRP) reduces adipocyte size and lipogenesis in eWAT. **(A)** Hematoxylin and eosin staining of the eWAT, followed by adipocyte size measurement. **(B)** Western blotting was used to examine representative pictures and relative band intensities for lipid metabolism enzymes such as fatty acid synthase (FAS), acetyl-CoA carboxylase (ACC), and carbohydrate-responsive element-binding protein. The intensity of the bands was calibrated to that of *β*-actin, and the mean percentage of ND ± standard error of the mean is shown in the bar figures. The letters (a–d) represent statistically significant differences (*p* < 0.05) assessed using Duncan’s multiple range test. NC, normal control diet; HF, high-fat diet; FOS, fructooligosaccharide.

AGRP administration significantly reduced the size of adipocytes in white adipose tissue compared to that in the HFD group (*p* < 0.05, Figure 5A), which was associated with a decrease in body weight and fat content (Figures 2, 4). In addition, as a result of the analysis of lipogenic proteins in white adipose tissue, the expression level of lipogenesis-related proteins in the AGRP administration group was significantly reduced compared to that in the HFD group (*p* < 0.05, Figure 5B), indicating that AGRP alleviated the symptoms of HFD-induced fatty liver disease.

### 3.8 AGRP mitigates intestinal inflammation and improves mucus production and intestinal permeability

**Figure 6:**
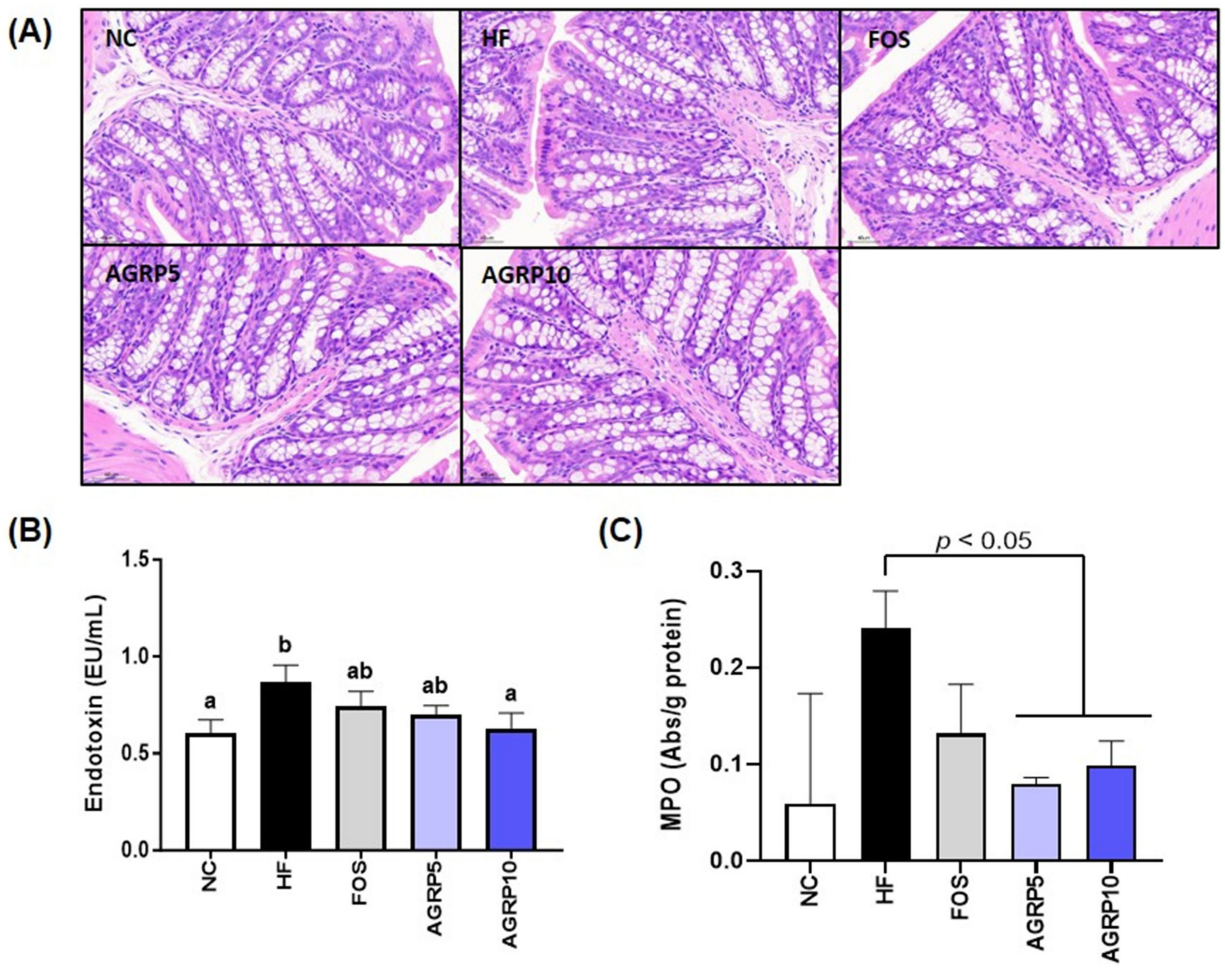
*Acorus gramineus* rhizome-derived polysaccharides (AGRP) reduces enterotoxins, alleviating intestinal inflammation and improving mucus production and permeability. **(A)** Representative histological results from hematoxylin and eosin (H&E) staining in colon tissue. **(B)** Endotoxin, **(C)** Myeloperoxidase (MPO). The data was shown as mean ± standard error of the mean (*n* = 9). The letters (a–d) represent statistically significant differences (*p* < 0.05) assessed using Duncan’s multiple range test. NC, normal control diet; HF, high-fat diet; FOS, fructooligosaccharide.

The reduction in intestinal inflammation and recovery of mucus-producing goblet cells in the AGRP group were confirmed by histological analysis of intestinal tissue stained with H&E at levels that were comparable to those in the ND group, but distinct from those in the HFD group (Figure 6A). Furthermore, compared to the HFD group, the AGRP10 group blood endotoxin levels decreased in a dose-dependent manner (Figure 6B). MPO activity, a biomarker of neutrophil infiltration and inflammation, was significantly elevated in the HFD group. AGRP administration significantly reduced MPO levels compared to the HFD group (*p* < 0.05), suggesting attenuation of intestinal inflammation. Our results suggest that AGRP extract may enhance mucus production and intestinal permeability by reducing metabolic endotoxemia.

## 4 Discussion

Chemical analysis of AGRP revealed a diverse composition of monosaccharides, predominantly galacturonic acid, followed by arabinose, galactose, xylose, and glucose (Figure 1; Table 1). Such monosaccharides are key structural units of polysaccharides commonly found in dietary fiber sources that exhibit prebiotic properties and metabolic health benefits. For instance, galacturonic acid and arabinose-rich polysaccharides have been reported to selectively stimulate the growth of beneficial gut bacteria, including *Bifidobacterium* and *Lactobacillus*, which are closely associated with enhanced SCFA production and improved metabolic regulation (7, 16–19). Additionally, galactose and xylose units are characteristic of arabinoxylans and arabinogalactans, which have demonstrated immunomodulatory and anti-obesity effects through microbial fermentation in the colon (7, 17, 18).

AGRP exhibited significant prebiotic activity, particularly at a concentration of 0.5%, promoting the growth of beneficial bacteria, such as *Lactobacillus* and *Bifidobacterium* (Figures 1A–C). *Lactobacillus bulgaricus* showed growth rates significantly higher compared to inulin, emphasizing AGRP’s superior prebiotic potential. AGRP-induced fermentation increased short-chain fatty acids (SCFAs), especially butyrate (Figure 1D), crucial for maintaining epithelial integrity, modulating immune responses, and reducing systemic inflammation (7, 19). Although oligosaccharides are primarily recognized as prebiotics, polysaccharides like AGRP exert similar benefits after enzymatic hydrolysis by gut microbiota (16, 19). Gut microbial glycoside hydrolases degrade polysaccharides into fermentable oligosaccharides, subsequently utilized by beneficial microbes. This microbial utilization supports intestinal homeostasis and metabolic responses, particularly under high-fat diet conditions (Figures 2–4) (20–25).

Butyrate, in particular, plays multiple essential roles in gut health and metabolism. It serves as a primary energy source for colonocytes, enhancing gut barrier integrity and preventing systemic endotoxin translocation and inflammation (20, 21). Butyrate also modulates immune responses, potentially lowering chronic inflammation, influences gut hormones regulating appetite and energy expenditure, and improves lipid metabolism and insulin sensitivity (22–25). Thus, AGRP-mediated butyrate enhancement may significantly contribute to metabolic health benefits.

Hepatic analysis of AGRP-treated mice confirmed reduced liver lipid accumulation compared to the HFD group (Figures 4A,B). Histological assessments using hematoxylin and eosin (H&E) staining showed pronounced hepatic lipid droplet accumulation characterized by numerous large lipid vacuoles in the HFD control group. In contrast, positive control and AGRP-treated mice exhibited markedly reduced lipid droplet size and density, demonstrating improvement in hepatic steatosis. Oil Red O staining further confirmed these results, revealing extensive lipid deposits stained intensely red in the control group, whereas lipid accumulation was notably reduced in positive control and AGRP-treated groups (Figure 4B). Consistently, Western blot analysis showed significant differences among groups in the expression of key lipogenic proteins including sterol regulatory element-binding protein 1c (SREBP-1c), fatty acid synthase (FAS), and acetyl-CoA carboxylase (ACC). The HFD control displayed elevated expression of these lipogenic markers, indicating enhanced hepatic lipogenesis. AGRP-treated groups significantly downregulated the expression of these proteins, reflecting suppression of lipogenic pathways and improved lipid metabolism (Figure 4C). Serum analyses demonstrated systemic benefits, including improved lipid profiles (total cholesterol, triglycerides, LDL-C) and enhanced glucose tolerance and insulin sensitivity (OGTT, HOMA-IR; Figure 3). These comprehensive metabolic improvements highlight AGRP’s ability to address multiple aspects of MASLD-related metabolic dysregulation (26).

Histological and molecular analyses of epididymal white adipose tissue (eWAT) further demonstrated the beneficial metabolic effects of AGRP treatment (Figures 5A,B). Epididymal adipose tissue is a major fat depot critically involved in lipid storage, systemic insulin sensitivity, and inflammation, and is significantly affected by obesity induced by a high fat diet. The histological analysis (Figure 5A) revealed that adipocytes in the HFD control group were notably enlarged, reflecting substantial adipocyte hypertrophy characteristic of impaired lipid metabolism and obesity associated inflammation. Conversely, adipocyte size and tissue hypertrophy were markedly reduced in the positive control and AGRP treated mice, indicative of improved adipocyte function and lipid handling. Western blot analyses of eWAT (Figure 5B) provided molecular level support for these histological observations by showing reduced relative expression (band intensities) of key lipogenic enzymes including fatty acid synthase (FAS), acetyl CoA carboxylase (ACC), and carbohydrate responsive element binding protein (ChREBP) in AGRP treated groups compared to the HFD control. Lower expression of these proteins confirms suppression of lipogenic pathways and adipogenesis, correlating with decreased adipocyte hypertrophy and improved lipid metabolism. Collectively, these histological and molecular findings clearly illustrate AGRP’s therapeutic role in attenuating adipose tissue dysfunction and systemic metabolic abnormalities associated with obesity.

The combined effects of AGRP on gut microbiota and hepatic lipid metabolism underscore its multifaceted therapeutic potential. AGRP exerts a synergistic effect on gut–liver axis dysfunction, crucial in MASLD progression. In the gut, clear histopathological differences were observed among the Normal Control (NC), high fat diet (HFD), and AGRP-treated groups (Figure 6A). The NC group exhibited intact intestinal structure characterized by well-organized crypts, abundant mucus-producing goblet cells, and minimal inflammatory cell infiltration, indicative of a healthy gut barrier. In contrast, the HFD group showed disrupted crypt architecture, significant depletion of goblet cells, and elevated infiltration of inflammatory cells into the mucosal layer, reflecting compromised intestinal integrity and increased inflammation associated with obesity-induced gut dysfunction. AGRP treatment markedly reversed these pathological changes by restoring crypt structure, increasing goblet cell numbers, and reducing inflammatory cell infiltration compared to the HFD group. Additionally, AGRP significantly reduced circulating endotoxin levels (Figure 6B). Furthermore, decreased MPO activity in colon tissue (Figure 6C) supports its role in alleviating intestinal inflammation under high-fat diet conditions. Collectively, these results emphasize AGRP’s beneficial effects on microbial composition and intestinal barrier integrity, thereby reducing systemic inflammation associated with metabolic disorders. Through simultaneous modulation of gut microbiome and hepatic lipid metabolism, AGRP effectively addresses the complex interplay underlying MASLD. These results are consistent with previous reports on the metabolic effects of plant-derived polysaccharides in high-fat diet-induced models. While studies on *Acorus gramineus* polysaccharides are limited, related compounds from *Rhizoma Acori Tatarinowii* have shown anti-obesity and lipid-lowering effects through gut microbiota modulation and improved insulin sensitivity (16, 17). Similar outcomes have also been observed with other dietary fibers such as gellan gum and molokhia leaf extract (14, 15), supporting the role of microbiota-targeted polysaccharides in metabolic regulation.

Taken together, AGRP offers several distinct advantages as a prebiotic compound. First, its rich content of galacturonic acid, arabinose, and galactose provides a favorable carbohydrate composition that supports the selective growth of beneficial gut bacteria such as *L. bulgaricus* and *B. longum*. Second, its fermentation significantly enhances butyrate production, exceeding the effects of inulin. Butyrate is a key short-chain fatty acid known to reinforce intestinal barrier function, reduce inflammation, and improve lipid and glucose metabolism. These combined properties not only promote gut health but also offer systemic metabolic benefits through modulation of the gut–liver axis. Thus, AGRP represents a promising and multifunctional dietary fiber that may surpass conventional prebiotics in therapeutic scope.

Building on these findings, future studies are warranted to determine the optimal dosing, long-term efficacy, and underlying molecular mechanisms of AGRP. In particular, clinical translation will benefit from expanded *in vivo* validation using various metabolic disease models and microbiome-targeted analyses, as well as human intervention trials to confirm AGRP’s functional role in metabolic health.

## 5 Conclusion

In conclusion, this study demonstrates that AGRP, a novel plant-derived polysaccharide from *A. gramineus* rhizome, exerts significant prebiotic and metabolic benefits by modulating the gut microbiota, enhancing butyrate production, and alleviating high-fat diet-induced metabolic dysfunction. AGRP promoted the growth of beneficial gut bacteria such as *Bifidobacterium* and *Lactobacillus*, improved metabolic health by reducing body weight gain, hepatic lipid accumulation, and insulin resistance, and ameliorated intestinal inflammation by strengthening gut barrier integrity. These effects were supported by reduced endotoxin levels, restored mucosal structures, and decreased MPO activity. Collectively, these findings indicate that AGRP has promising potential as a functional dietary intervention for the management of metabolic disorders, including MASLD. Future studies are warranted to explore optimal dosage and validate these outcomes in clinical settings.

## Data Availability

The datasets presented in this study can be found in online repositories. The names of the repository/repositories and accession number(s) can be found in the article/Supplementary material.
