# Supplementary material for: Prebiotic potential and metabolic benefits of Acorus gramineus rhizome-derived polysaccharides in a high-fat diet-induced obesity model
Source: Front Nutr. 2025 Jun 10;12:1605201. doi: 10.3389/fnut.2025.1605201 (PMC12185287; doi:10.3389/fnut.2025.1605201)
Supplement: Supplementary file 1 [file Table_1.docx]

Supplementary Material

**Table S1**. Multiple Reaction Mode (MRM) transitions for butyric acid quantification by GC-MS/MS

| Compound | RT (min) | DT (ms) | ion pairs* | CE (ev) | ion pairs** | CE(ev) |
| --- | --- | --- | --- | --- | --- | --- |
| Butyric acid | 11.50 | 10 | 60→43 | 10 | 73→55 | 5 |
| Butyric acid-1-13C  (Internal Standard) | 11.50 | 10 | 61→43 | 10 | 74→55 | 10 |

^*^Quantitative ion, ^**^Qualitative ion.

CE, collision energy; DT, dwell time; IS, internal standard; RT, retention time.
